# What's your neural function, visual narrative conjunction? Grammar, meaning, and fluency in sequential image processing

**DOI:** 10.1186/s41235-017-0064-5

**Published:** 2017-05-24

**Authors:** Neil Cohn, Marta Kutas

**Affiliations:** 1 Center for Research in Language, University of California, San Diego, La Jolla, CA USA; 2 Tilburg Center for Cognition and Communication, Tilburg University, P.O. Box 90153, 5000 LE Tilburg, the Netherlands; 3 Department of Cognitive Science, University of California, San Diego, La Jolla, CA USA

**Keywords:** Visual language, Visual narrative grammar, Discourse, Comics, P600, Left anterior negativity

## Abstract

Visual narratives sometimes depict successive images with different characters in the same physical space; corpus analysis has revealed that this occurs more often in Japanese manga than American comics. We used event-related brain potentials to determine whether comprehension of “visual narrative conjunctions” invokes not only incremental mental updating as traditionally assumed, but also, as we propose, “grammatical” combinatoric processing. We thus crossed (non)/conjunction sequences with character (in)/congruity. Conjunctions elicited a larger anterior negativity (300–500 ms) than nonconjunctions, regardless of congruity, implicating “grammatical” processes. Conjunction and incongruity both elicited larger P600s (500–700 ms), indexing updating. Both conjunction effects were modulated by participants’ frequency of reading manga while growing up. Greater anterior negativity in frequent manga readers suggests more reliance on combinatoric processing; larger P600 effects in infrequent manga readers suggest more resources devoted to mental updating. As in language comprehension, it seems that processing conjunctions in visual narratives is not just mental updating but also partly grammatical, conditioned by comic readers’ experience with specific visual narrative structures.

**Electronic supplementary material:**

The online version of this article (doi:10.1186/s41235-017-0064-5) contains supplementary material, which is available to authorized users.

## Significance

Sequential images are ubiquitous in contemporary society, and their assumed transparency has made them popular in instruction manuals and as experimental stimuli, beyond their role in entertainment like comics. Such uses assume that sequential image understanding involves basic perceptual and/or semantic processing, which are uniform across individuals. Recent work, however, argues that sequential images use a “narrative grammar” that varies in different cultural contexts. By examining a cultural pattern that occurs more often in Japanese manga than in American comics, we show herein that processing does not solely and uniformly rely on semantic updating, and is modulated by experience with those patterns (i.e., reading manga). Our results imply that sequential images are not as transparent or uniform as presumed, which raises questions of how universally accessible they truly are as experimental stimuli and educational materials.

## Background

Drawn sequential images are ubiquitous in human communication; they extend throughout human history and across cultures from cave paintings and scrolls to contemporary comics and storyboards that guide storytelling in films (McCloud, 1993). In science, sequential images are popular as experimental stimuli in studies of theory of mind (Baron-Cohen, Leslie, & Frith, 1986; Sivaratnam, Cornish, Gray, Howlin, & Rinehart, 2012), event sequencing (Tinaz, Schendan, Schon, & Stern, 2006), and cross-cultural temporal cognition (Núñez & Cooperrider, 2013), among others. Image sequencing tasks are also staples within IQ assessment (Kaufman & Lichtenberger, 2006; Ramos & Die, 1986), and a growing movement has advocated using visual narratives such as comics in education (Short, Randolph-Seng, & McKenny, 2013). This prevalence of sequential images is underlined by a belief that their comprehension is not only universal but also fairly transparent (Berliner & Cohen, 2011; Levin & Simons, 2000; McCloud, 1993). Given these diverse real-world contexts, we ask: how uniform is visual narrative processing?

These universality and transparency assumptions are inherent in a common theoretical framework for visual narrative processing on which comprehenders dynamically update their mental model of a scene as they view successive images. Comprehension thus proceeds via incremental updating of a mental representation based on perceptual (Berliner & Cohen, 2011; Levin & Simons, 2000) and/or semantic analysis of each panel in the sequence (Bateman & Wildfeuer, 2014; Magliano & Zacks, 2011; McCloud, 1993). This presumes that sequential image comprehension engages basic cognitive processing (perceptual and semantic systems) which operates similarly across individuals.

### *Visual Narrative Grammar*

Despite its prevalence and seeming transparency, a growing literature suggests that visual narrative processing may be more complex than this framework implies. *Visual Narrative Grammar* (VNG), in particular, proposes that, in addition to updating perceptuo-semantic information, sequential image comprehension involves a hierarchical narrative grammar, and that these updating and grammatical processes interact (Cohn, 2013b). VNG assigns narrative categories to panels (Cohn, 2014b), organized into hierarchical constituents (Cohn, Jackendoff, Holcomb, & Kuperberg, 2014). This narrative grammar functions as part of the textbase to package semantic information which in turn is incorporated into a situation model of visual discourse (Van Dijk & Kintsch, 1983; Zwaan & Radvansky, 1998). Because of this, narrative structure operates via a processing stream distinct from that for semantics (Cohn, Paczynski, Jackendoff, Holcomb, & Kuperberg, 2012a), and is indexed by different neural markers (Cohn et al., 2014; Cohn, Paczynski, et al., 2012a). As argued elsewhere, the processes involved in comprehending visual narratives are analogous to those involved in sentence processing (Cohn et al., 2014; Cohn, Paczynski, et al., 2012a; Magliano, Larson, Higgs, & Loschky, 2015)—including those for structural aspects (syntax), meaning, and their interaction (Jackendoff, 2002)—as indexed by ostensibly similar neural mechanisms for sentences and visual narratives (e.g., Friederici, 2011; Hagoort, 2003), as discussed in the following. In this report, we investigate the neural processing of a particular, presumably grammatical, construction in sequential visual narratives—conjunction—to further test this aspect of VNG, and to determine whether such processing is modulated by participants’ experience with comics in which this construction is more or less prevalent.

In VNG, a basic sequence is composed of a canonical narrative pattern (Cohn, 2013b). Establishers set up a situation, often followed by Initials, which depict the start of the events relevant for the narrative sequence. The sequence climaxes in a Peak, with an aftermath or resolution occurring in a Release. These categorical roles are assigned as a function of a panel’s semantic content (i.e., the meaningful cues depicted in the image) and its context in a global sequence (Cohn, 2013b, 2014b). A canonical constituent is comprised of these core categories in this order, a preference which persists in motion graphics (Barnes, 2017) and film (Amini, Riche, Lee, Hurter, & Irani, 2015). Narrative categories apply to both the panel level and the constituent level; that is, just like individual panels, whole groupings of panels can play particular narrative roles. An Arc is a constituent that plays no role in a larger structure.

**Fig. 1:**
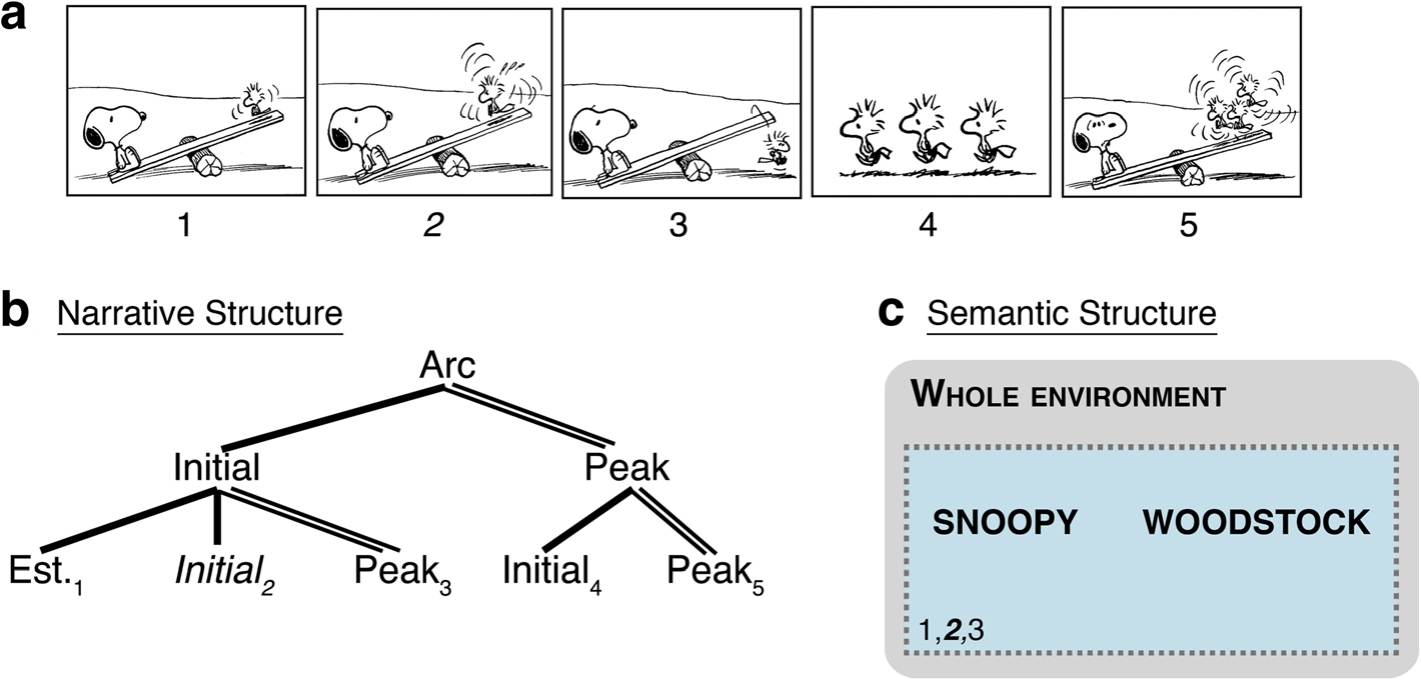
(**a**) Visual narrative sequence that uses (**b**) a simple hierarchic narrative structure which (**c**) maps to a spatial semantic structure

Figure 1a depicts a sequence of Woodstock and Snoopy playing on a teeter-totter. As diagrammed in Fig. 1b, the sequence first sets up the situation (Establisher) and then shows Woodstock struggling on the teeter-totter (Initial). This action climaxes in a Peak, where he jumps off. He then recruits friends to help him in another Initial, which sets up a climactic final Peak. A simple constituent structure emerges from this sequence, with Woodstock’s struggles providing the overall “set up” (Initial constituent) for the overall climax of his recruiting friends (Peak constituent).

Figure 1c also diagrams spatial aspects of the semantic structure in the first three panels (diagramming of other structures remains omitted). The first three panels all show the scene with both Snoopy and Woodstock, and thus the spatial structure includes this whole viewpoint (depicted with the dotted line). Panel numbers in Fig. 1 correspond to the indices linking these structures throughout a parallel architecture (Cohn, 2015; Jackendoff, 2002).

**Fig. 2:**
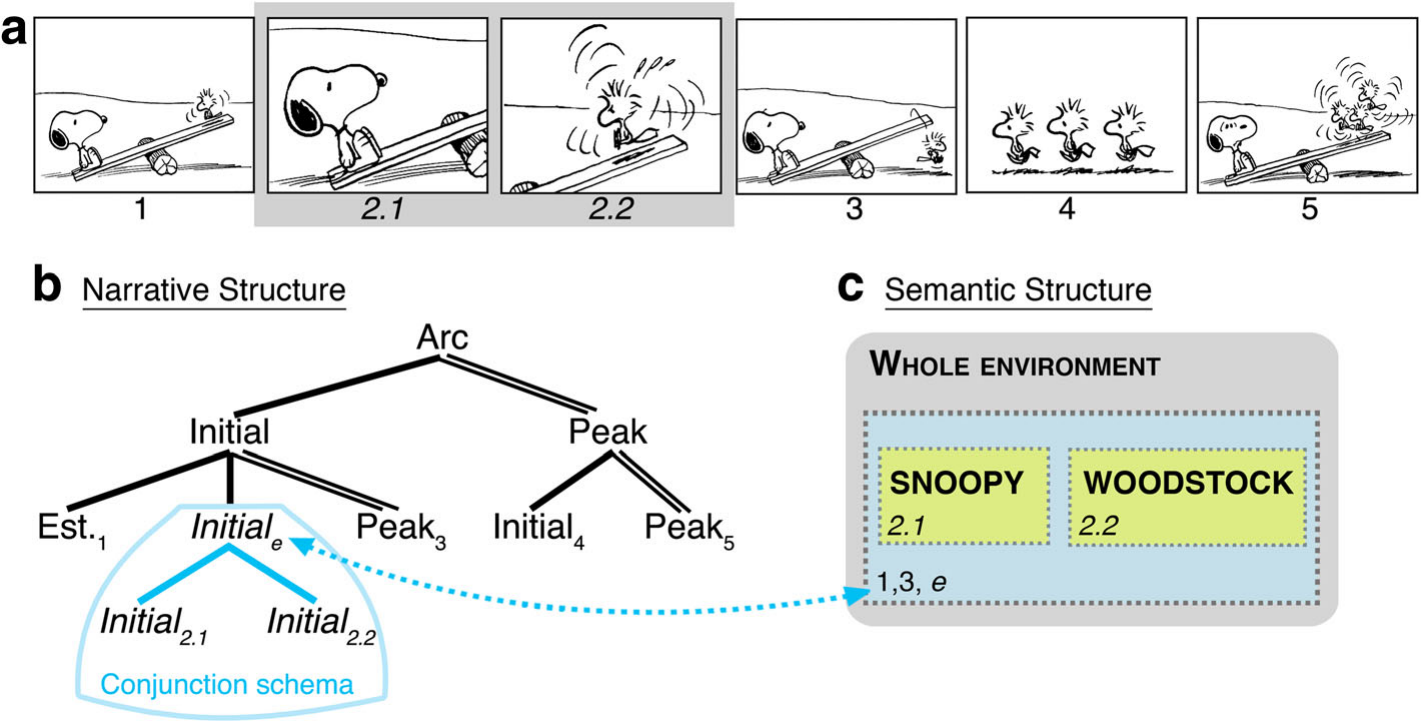
(**a**) Visual narrative sequence where single characters are framed in separate panels, causing (**b**) the narrative structure to use using E(nvironmental)-Conjunction, which (**c**) maps to a semantic structure requiring a spatial inference

Now consider Fig. 2a. Here, Snoopy and Woodstock appear in separate, successive images (Fig. 2a, panels 2.1 and 2.2), rather than in a single image as in Fig. 1a, panel 2. Comprehension of these panels requires inferring a larger spatial environment (Fig. 2c, “e”) because both characters belong in the same space, despite their appearance in separate panels. Indeed, a single image could readily show this same information (Fig. 1a, panel 2), obviating the need for an inference and consequent mental updating.

VNG posits that comprehenders draw this common-space inference and use hierarchic, combinatoric structures separate from, yet interfacing with, the updating of the perceptuo-semantic content of these panels to understand the visual narrative (Cohn, 2013b, 2014a, 2015; Cohn, Paczynski, et al., 2012a). In Fig. 1a, the Initial (panel 2) depicts Woodstock unsuccessfully bouncing on a teeter-totter. Functionally, this information sets up Woodstock’s action of hopping off in the next panel (Peak). When this image is divided into two panels (Fig. 2a), VNG assigns both the same narrative role within a “conjunction schema” sharing that category (Fig. 2b). This is structurally analogous to syntactic conjunction in language, where a phrase repeats the same syntactic category, as in [_*NP*_[_*N*_ *Snoopy*] *and* [_*N*_ *Woodstock*]], a noun phrase with two nouns.

In the version in Fig. 2a both panels act as Initials, conjoined within an Initial constituent (diagrammed in Fig. 2b). This narrative information interfaces with semantic content (Fig. 2c), such that each Initial contains one character (indexed across structures; Fig. 2a, panels 2.1 and 2.2), and their inferred union (Fig. 2a, “e”) maps to the whole constituent (dotted blue line). VNG calls this construction *E(nvironmental)-Conjunction*: it is a *narrative* conjunction that maps to an inferred *semantic* environment. Several types of semantic information other than spatial inference can also map to narrative conjunction schemas (Cohn, 2015).

### Processing of visual narratives

Research on visual narrative processing using event-related brain potentials (ERPs) has supported interactions between retrieval and integration/updating mechanisms, as in discourse theories (Van Dijk & Kintsch, 1983; Zwaan & Radvansky, 1998). Comprehenders access the meaning of an image relative to its sequential context, as indexed by the N400 (Kutas & Federmeier, 2011), a negative-going deflection peaking roughly 400 ms after stimulus onset and, for images, typically with a widespread anterior scalp distribution (Barrett, Rugg, & Perrett, 1988; Holcomb & McPherson, 1994; West & Holcomb, 2002). The N400 has been interpreted as a default brain response indexing the retrieval of semantic information for a stimulus given preceding context (Brouwer & Hoeks, 2013; Kutas & Federmeier, 2011). As a result, larger N400s are observed to unexpected or anomalous aspects of individual objects or scenes (Võ & Wolfe, 2013), images in visual narratives (Cohn, Paczynski, et al., 2012a; West & Holcomb, 2002), and events in video sequences (Amoruso et al., 2013; Reid & Striano, 2008; Sitnikova, Kuperberg, & Holcomb, 2003), just as to unexpected or anomalous words in sentence contexts (Kutas & Federmeier, 2011; Kutas & Hillyard, 1980).

The information integration or updating has been linked to posterior positivities such as the P600, beginning around 400–500 ms (Brouwer, Fitz, & Hoeks, 2012; Donchin & Coles, 1988; Kuperberg, 2013). In linguistic contexts, the P600 was first tied to syntactic violations (Hagoort, Brown, & Groothusen, 1993; Osterhout & Holcomb, 1992), but was later also associated with nonsyntactic thematic role violations (Kuperberg, Sitnikova, Caplan, & Holcomb, 2003), humor (Coulson & Kutas, 2001), and nonverbal violations in music (Patel, Gibson, Ratner, Besson, & Holcomb, 1998) and sequence learning (Christiansen, Conway, & Onnis, 2011). In the visual domain, P600s have been elicited by situational changes in visual narratives (Cohn & Kutas, 2015), violations to the internal components of scenes and/or events (Cohn & Maher, 2015; Sitnikova, Holcomb, & Kuperberg, 2008; Võ & Wolfe, 2013), and groupings of panels into ill-formed narrative constituents (Cohn et al., 2014). Given these diverse findings, the P600 has subsequently been associated with the prediction error generated from a discontinuity with a prior context, resulting in the alteration or updating of a mental model related to semantics or structure (Brouwer et al., 2012; Donchin & Coles, 1988; Kuperberg, 2013).

This updating process is consistent with discourse theories positing that readers incur a cost for updating discontinuities of referential, spatial, and/or event information in constructing a situation model of a discourse (Van Dijk & Kintsch, 1983), as in the event*-*indexing model (Radvansky & Zacks, 2014; Zwaan & Radvansky, 1998). Indeed, P600s have been evoked by unexpected, novel, or ambiguous referential information (mismatching pronouns or character changes) in a discourse context (Burkhardt, 2006, 2007; Ferretti, Rohde, Kehler, & Crutchley, 2009; Nieuwland & Van Berkum, 2005; Van Berkum, Koornneef, Otten, & Nieuwland, 2007) and by changes in characters and/or events in visual narratives (Cohn & Kutas, 2015). The event horizon model further argues that these situational changes also cue segmentation (Gernsbacher, 1990; Radvansky & Zacks, 2014). Here, the prediction error from situational changes marks a boundary between constituents, triggering the updating process (Radvansky & Zacks, 2014; Zacks, Speer, & Reynolds, 2009), as suggested by behavioral and/or neurocognitive measures aligned with participants’ identification of boundaries between events and/or discourse segments (Magliano & Zacks, 2011; Zacks et al., 2001, 2009).

Situational changes alone, however, cannot account for constituent structure in visual narratives. VNG’s narrative categories are more predictive of segmentation choices in drawn visual narratives than semantic situational changes (Cohn & Bender, 2017), and P600s also differ between sequences in which inferential situational change is held constant but narrative structure differs (Cohn & Kutas, 2015). In addition, while backward-looking updating processes have been observed to disruptions following a narrative constituent break, a different ERP effect—an anterior negativity (left lateralized and right prefrontal)—contrasts disruptions that *precede* the boundary between constituents (Cohn et al., 2014). Such effects suggest forward-looking combinatoric processes which could not be captured by an updating process. Anterior negativities appear to be sensitive to combinatoric processing of VNG, but not to semantics. Cohn, Paczynski, et al. (2012a), for example, observed a left-lateralized anterior negativity to panels in scrambled sequences compared with those with a coherent narrative structure, absent of semantic relations between the images (analogous to sentences like *Colorless green ideas sleep furiously*, which use syntax but no semantic relationships between words). By contrast, narrative structure, in the absence of semantic associations between panels, did not attenuate the semantically sensitive N400. This pattern of effects was taken to suggest that narrative structure and semantics operated on different processing streams.

These findings in visual narratives are reminiscent of the left anterior negativities (LAN) between 300 and 500 ms elicited by syntactic violations in language (e.g., Friederici, 2011; Hagoort, 2003; Neville, Nicol, Barss, Forster, & Garrett, 1991), where they have been interpreted as indices of violated structural expectations (Hoen & Dominey, 2000; Lau, Stroud, Plesch, & Phillips, 2006). Similar anterior negativities with rightward lateralization (RAN) have been observed in response to “syntactic” violations during music processing (Koelsch, Gunter, Wittfoth, & Sammler, 2005; Patel et al., 1998). The similarities among these anterior negativities in language, music, and visual narratives have led to speculation that they index a common, domain-general mechanism for combinatoric (grammatical) processing (Cohn et al., 2014; Patel, 2003).

In light of the extant electrophysiological literature, VNG predicts two distinct ERP effects in response to E-Conjunction: a P600 indexing the cost of integrating two separate characters into a single mental model and/or revising structures; and an anterior negativity indexing the combinatoric processes of the narrative grammar, which we take to be independent of the processing of situational changes (elaborated later).

### Cross-cultural variation

Because VNG is embedded in a paradigm that posits different cultural “visual languages” (Cohn, 2013a), it predicts that E-Conjunction processing will be modulated by the extent of a comprehender’s experience with visual narratives containing this construction. Our corpus analysis revealed that on average Japanese manga contains more E-Conjunction than American comics (Cohn, 2011, 2013a, in press; Cohn, Taylor-Weiner, & Grossman, 2012b). Accordingly, we might expect differences in E-Conjunction processing between manga readers, who likely store these schematic structures as part of their “visual language” fluency, and readers of American comics, for whom such structures are less entrenched. Some role for experience is suggested by findings that naïve film viewers from a remote Turkish village have deficits generating “spatial inferences” from films using sequences akin to E-Conjunction (Ildirar & Schwan, 2015; Schwan & Ildirar, 2010). While this effect of experience held for individuals lacking exposure to visual narratives, we would expect processing differences within experienced comic readers based on which comics they have read. Such differences would extend beyond basic fluency effects due to general comic reading expertise (Nakazawa, 2016), including those observed in ERP amplitude modulation (Cohn & Kutas, 2015; Cohn & Maher, 2015; Cohn, Paczynski, et al., 2012a).

### The current study

**Fig. 3:**
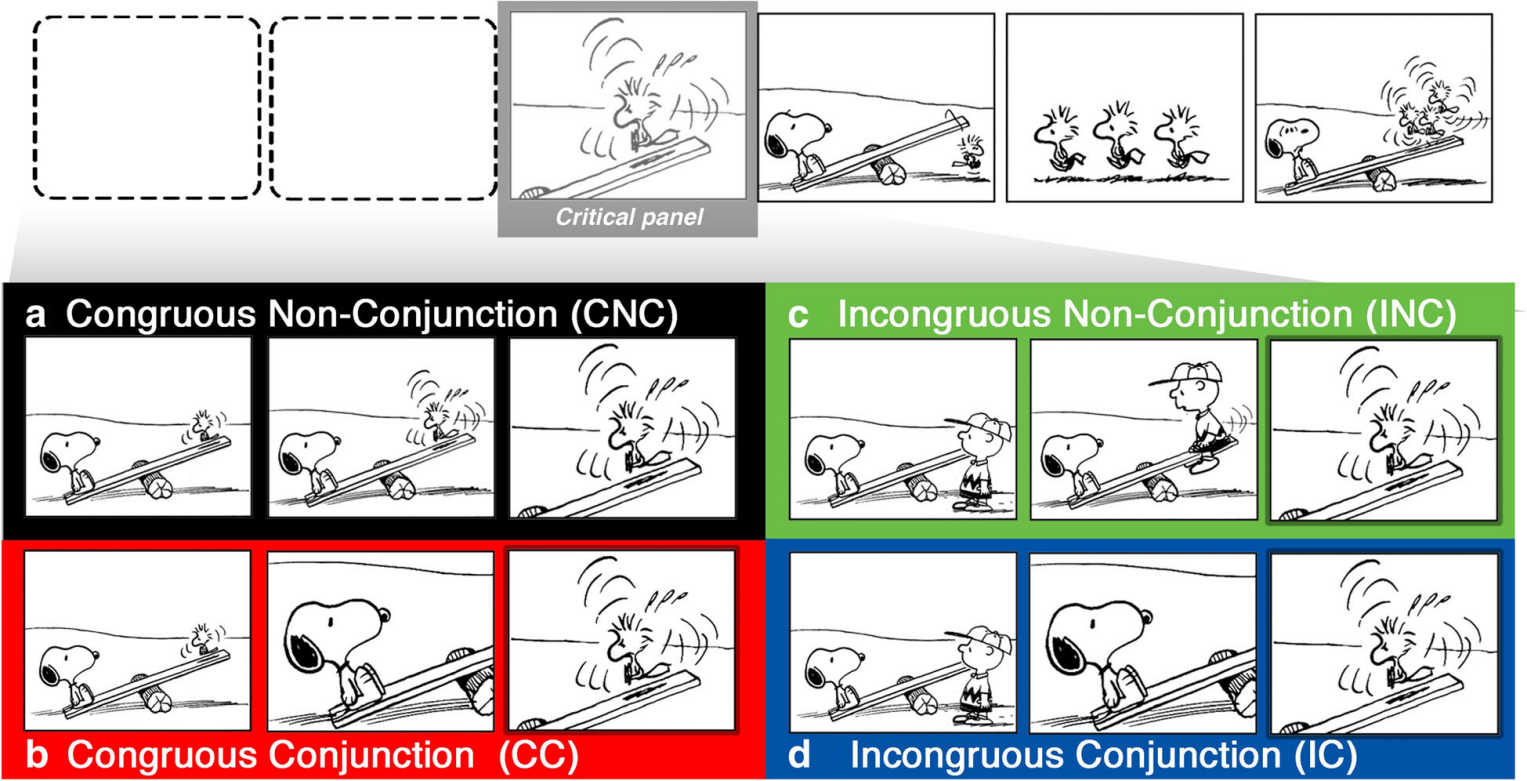
Experimental sequence types crossing Conjunction with Congruity. **a** Congruous Non-Conjunction (CNC). **b** Congruous Conjunction (CC). **c** Incongruous Non-Conjunction (INC). **d** Incongruous Conjunction (IC)

In the current study we manipulated narrative conjunctions to help adjudicate between the view that visual narrative processing relies on meaning-based relationships between images feeding incremental mental updating (Magliano & Zacks, 2011; Radvansky & Zacks, 2014) or on generic perceptual processes to account for spatial coherence across film shots (Berliner & Cohen, 2011; Levin & Simons, 2000), and VNG which posits an additional combinatoric narrative grammar component, independent of semantics, as well. To that end, we crossed (non)conjunction sequences with (in)congruity where characters either did or did not change midway through the sequence (Fig. 3).

By all accounts, referential changes and spatial inference triggered by conjunctions would both be indexed by a P600, reflecting updating of a mental model (Brouwer et al., 2012; Donchin & Coles, 1988). Updating processes for discontinuities created by characters changing midway through the sequence would be consistent with P600s evoked by referential change in verbal discourse (e.g., Nieuwland & Van Berkum, 2005; Van Berkum et al., 2007) and visual narratives (Cohn & Kutas, 2015). Conjunctions should lead to updating because separately depicted characters would be integrated into a single spatial environment, and/or because a surface change in characters should generate prediction error, as in the event horizon model (Radvansky & Zacks, 2014). P600s to conjunction could also reflect the revision or updating of narrative structural constraints given a prior context (Cohn et al., 2014; Cohn & Kutas, 2015), as occurs in the reanalysis of syntactic parsing (Osterhout & Holcomb, 1992), including for conjunctions (Brown & Hagoort, 2000; Brown, Hagoort, & Kutas, 2000) and nonconceptual music (Patel et al., 1998). Thus, co-occurrence of conjunction with discontinuity may demand an even more substantive updating process to reconcile the structural revision and/or spatial inference with the semantic incongruity.

Moreover, VNG further posits an anterior negativity reflecting engagement of the narrative grammar for conjunction processing. We expect these processes to be insensitive to semantic congruity like the discontinuity of character changes. Such results also would be consistent with reports of P600s to parsing ambiguities between phrasal and sentence-level conjunctions in sentences (Brown & Hagoort, 2000; Brown et al., 2000), and both LAN and P600s to conjunctions in the context of ungrammatical or nonpreferred grammatical continuations of syntactic ambiguities (Kaan & Swaab, 2003).

Last but not least, VNG further predicts modulation of these ERP effects by participants’ experience with visual narratives containing conjunctions. Theories of situational semantic and/or perceptual models may predict variation with differing world knowledge (Hagoort, Hald, Bastiaansen, & Petersson, 2004) or construal based on different sociosemiotic contexts (Bateman & Wildfeuer, 2014), but we predict modulation purely on the basis of exposure to narrative. Accordingly, we expect experience to modulate conjunction-related processing, but not necessarily situational discontinuity.

## Methods

### Stimuli

We created 100 sequences 5–7 panels in length using wordless images from *The Complete Peanuts* by Charles Schulz (1952–1974), as in prior research (e.g., Cohn et al., 2014; Cohn & Maher, 2015; Cohn, Paczynski, et al., 2012a). Sequences began with at least one panel introducing both characters within the same spatial environment, as in Fig. 3. Congruous Non-Conjunction (CNC) sequences then showed both characters again in an initiating state (Initial), followed by a critical panel “zooming in” on only the second character (Fig. 3a). Congruous Conjunction (CC) sequences divided this Initial panel, by showing the first character in one panel and the second character in the subsequent critical panel (Fig. 3b). Incongruous sequences started by substituting a different character for the one ultimately appearing in the critical panel. Incongruous Non-Conjunction (INC) sequences began with different characters, which then changed in the critical panel (Fig. 3c), while Incongruous Conjunction (IC) sequences began with two characters, showed the first character, and then switched in the critical panel (Fig. 3d). Thus, the same critical panels appeared across all sequence types, either at the third or fourth position in the sequence. Sequences were counterbalanced in a Latin Square Design into four lists such that no list repeated strips. One hundred filler sequences featured varying degrees of coherence to further increase the heterogeneity of the stimuli and reduce the possibility of participants detecting our experimental manipulations.

### Participants

We recruited 28 self-described “comic readers” (12 male, 16 female, mean age: 20.9) from University of California, San Diego, USA. All participants were right-handed English speakers with normal vision, and gave informed written consent according to the UCSD Human Research Protections Program. Each participant completed the Visual Language Fluency Index (VLFI) questionnaire (Cohn, Paczynski, et al., 2012a) used to assess their expertise in understanding visual narratives. Expertise was operationalized as participants’ self-rated frequency of reading comic books, comic strips, graphic novels, and Japanese manga, as well as drawing comics, both currently and while growing up (1 = never, 7 = always). They also rated their self-assessed “expertise” at reading and drawing comics (1 = below average, 5 = above average). These ratings were combined to compute a “VLFI score” for each participant, which has consistently correlated with ERP indices of visual narrative processing (Cohn & Kutas, 2015; Cohn & Maher, 2015; Cohn, Paczynski, et al., 2012a) as well as with various behavioral measures (Cohn & Bender, 2017; Cohn & Wittenberg, 2015; Hagmann & Cohn, 2016); these ERP studies had sample sizes consistent with those examined here (i.e., 24–36 participants). An idealized average VLFI score falls around 12, a low score below 7 and a high score above 20. Participants’ mean fluency was a high average of 17.82 (SD = 6.4, range: 8.25–35.25).

These prior findings examined an aggregated VLFI score as a proxy for “fluency” for understanding sequential images in general. However, because we were interested in participants’ specific comic reading habits rather than their aggregate “fluency,” we focused on the components of the VLFI (Table 1). We did not, however, screen participants for readership of specific types of comics.

**Table 1:** Mean ratings for participants’ self-assessed reading frequency for various types of visual narratives

Type of experience	Currently (range)	While growing up (range)
Comic books	3.46 (1–7)	4.7 (1–7)
Comic strips	3.34 (1–7)	4.63 (1–7)
Graphic novels	2.93 (1–6)	3.57 (1–7)
Japanese manga	3.54 (1–7)	4.86 (1–7)
Drawing comics	1.71 (1–5)	2.82 (1–6)

Scale: 1 = never read, 7 = always read

### Procedure

Participants sat in a comfortable chair facing a computer screen in a room separate from the experimenter and computers. Trials began with a screen reading “READY,” at which point participants pressed a button to begin. After a fixation cross, each panel of the sequence appeared in the center of the screen one at a time for 1350 ms. A 300-ms ISI prevented images from appearing animated. After each sequence concluded, a question mark prompted participants to rate the comprehensibility of each strip with “good” and “bad” rating buttons held in each hand (rotated between the right and left hands across participants and lists), as in prior research (Cohn & Kutas, 2015). A short practice list acclimated participants to the procedure. A post-test questionnaire assessed their conscious observations of the stimuli.

### Data analysis

We analyzed participants’ comprehensibility judgments (whether or not the sequence made sense) for each sequence type (CNC, CC, INC, IC) and each participant, and subjected these data to a 2 (Structure: Conjunction vs. Non-Conjunction) × 2 (Congruence: Congruous vs. Incongruous) repeated-measures ANOVA.

**Fig. 4:**
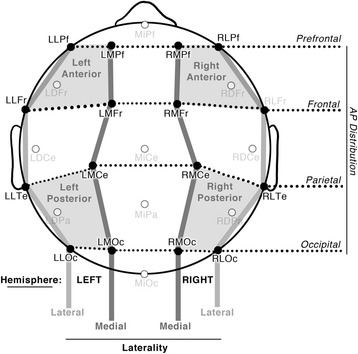
Electrode montage, illustrating 16 electrode sites analyzed across Hemisphere, Laterality, and Anterior–Posterior (*AP*) Distribution, as well as Quadrants used in follow-up analyses. *Fr* frontal, *L* left, *L* lateral, *M* medial, *Oc* occipital, *Pa* parietal, *Pf* prefrontal, *R* right, *Mi* Midline, *Ce* Central, *Te* Temporal

EEG was recorded from 26 tin electrodes evenly distributed across the scalp in a quasi-geodesic design (Fig. 4) referenced online to the left mastoid and re-referenced offline to the average of the right and left mastoids. Eye movements and blinks were monitored using electrodes placed beneath and next to each eye. Impedances were kept below 5 kΩ for all electrodes. EEG was digitized at a sampling rate of 250 Hz and bandpass filtered between 0.01 and 100 Hz with James Long amplifiers (www.JamesLong.net).

We analyzed ERPs time-locked to the onset of the critical panels across sequence types, and averaged within each sequence type across a 1500-ms epoch, relative to a 500-ms prestimulus baseline. Rejected EEG trials included those with eye blinks, eye movements, artifact caused by muscle movements, and/or artifact caused by signal loss or blocking (i.e., a flat line), assessed by visually inspecting raw data for each participant. Rejection rates were kept below 15% for each sequence type per participant. Trials retained after the artifact rejection process were used in our averaged ERP analysis.

We examined ERPs to the critical panel in the binned epochs of 300–500 ms, 500–700 ms, and 700–900 ms. Our omnibus within-subjects ANOVA looked for main effects and interactions of Structure (Conjunction vs. Non-Conjunction) and Congruence (Congruous vs. Incongruous) across 16 electrode sites that evenly divided eight electrodes each into factors of Hemisphere (left, right), Anterior–Posterior Distribution (prefrontal, frontal, parietal, and occipital) and Laterality (lateral, medial), as depicted in Fig. 4. We used a Bonferroni correction for multiple comparisons.

To investigate the effect of comic reading experience, we calculated the mean amplitude of the Conjunction minus Non-Conjunction sequences (collapsed across congruity) and averaged across all 16 electrodes from all four quadrants of the scalp (Fig. 4). We used a logistic regression to analyze these means by setting participants’ frequency ratings for reading habits of specific types of comics as predictors (see Table 1). We performed the same analysis for congruency (Incongruous minus Congruous). We followed significant findings by again running our ANOVA, but also including the measurements for any significant predictors as covariates.

## Results

### Behavioral results

Participants’ assessments of comprehensibility showed a main effect of congruity, *F*(1,27) = 20.72, *p* < 0.001: incongruous sequences were viewed as less comprehensible than congruous ones. A main effect of Structure, *F*(1,27) = 5.8, *p* < 0.05, and a Structure × Congruence interaction, *F*(1,27) = 8.6, *p* < 0.01, arose because Incongruous Non-Conjunction sequences (M = 0.58, SD = 0.11) were less comprehensible than Incongruous Conjunction sequences (M = 0.65, SD = 0.1). However, no difference in comprehensibility appeared between Congruous Non-Conjunction (M = 0.81, SD = 0.07) and Congruous Conjunction (M = 0.80, SD = 0.07) sequences. In posttest questionnaires, 61% of participants (17 of 28) without prompting noted that characters disappeared/changed in the sequence (i.e., congruous vs. incongruous). No participants explicitly distinguished Conjunction and Non-Conjunction sequences.

### Event-related potentials

**Fig. 5:**
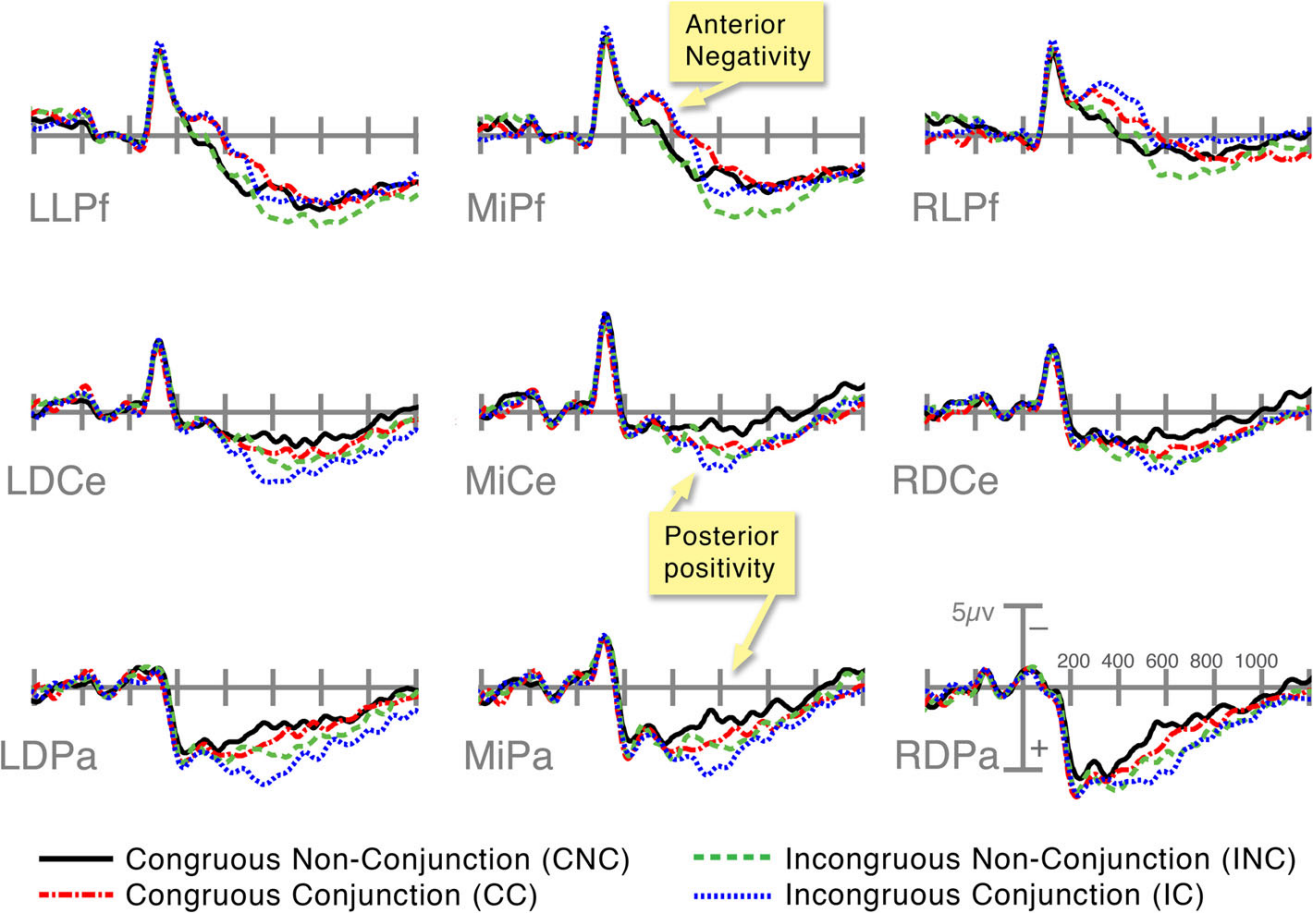
Illustration of grand-averaged ERPs time-locked to the critical panel across all sequence types at Prefrontal, Central, and Parietal electrode sites. *L* left, *L* lateral, *Pa* parietal, *Pf* prefrontal, *R* right, *Mi* Midline, *Ce* Central

Our analysis of the ERPs found several distinct patterns of effects: an anterior negativity between 300 and 500 ms and a more posteriorly distributed positivity extending from 400 through 900 ms. In the 300–500 ms epoch, panels in Conjunction sequences were more negative in anterior regions than those in Non-Conjunction sequences, regardless of congruity; this negativity peaked around 300 ms (see Fig. 5). This was suggested by a four-way interaction between Structure, Hemisphere, AP Distribution, and Laterality, *F*(3,81) = 15.71, *p* < 0.001.

A posterior positivity (P600) began around 400 ms and lasted past 900 ms (see Fig. 5), peaking near 550 ms. Conjunctions were more positive than Non-Conjunctions, and, with this same pattern, Incongruities were more positive than Congruities. This effect had a slight left posterior distribution. The start of this posterior effect in the 300–500 ms epoch was suggested by an interaction by Congruence and AP Distribution, *F*(3,81) = 3.98, *p* < 0.05. In both the 500–700 ms and 700–900 ms epochs, we found a main effect of Congruence (all *F* > 6.4, all *p* < 0.05), along with interactions between Congruence and Laterality (all *F* > 4.2, all *p* < 0.051), and between Structure, Hemisphere, AP Distribution, and Laterality (all *F* > 5.4, all *p* < 0.005). In the 700–900 ms epoch, we also found an interaction between Congruence and Structure with AP Distribution, *F*(3,81) = 6.6, *p* < 0.01, and Laterality, *F*(3,81) = 4.6, *p* < 0.05.

### Individual differences

To examine the effect of comic reading experience on conjunction processing, we compared the responses to the panels in Conjunction and Non-Conjunction sequences collapsed across congruity after averaging the amplitudes across all four scalp quadrants. Regression analysis for the 300–500 ms epoch indicated that the frequency of reading Japanese manga while growing up was the only reliable predictor (*β* = –0.57, *p* < 0.05); no other predictor approached significance (all *p* > 0.183). The overall model fit was *R* ^2^ = 0.61. An analysis of the variance inflation factors (VIF) showed that no predictor exceeded the recommended level of 10 (all VIF < 3.29), suggesting no confounding of multicollinearity.

A similar regression analysis in the 300–500 ms epoch collapsed across Congruence (Incongruous minus Congruous). We found only a trending predictor of frequency of comic strip reading while growing up (*β* = –0.36, *p* = 0.054), but no significance for manga reading either currently (*p* = 0.845) or while growing up (*p* = 0.915). In addition, neither Conjunction (*p* = 0.213) nor Congruence (*p* = 0.736) correlated with the general VLFI scores.

**Fig. 6:**
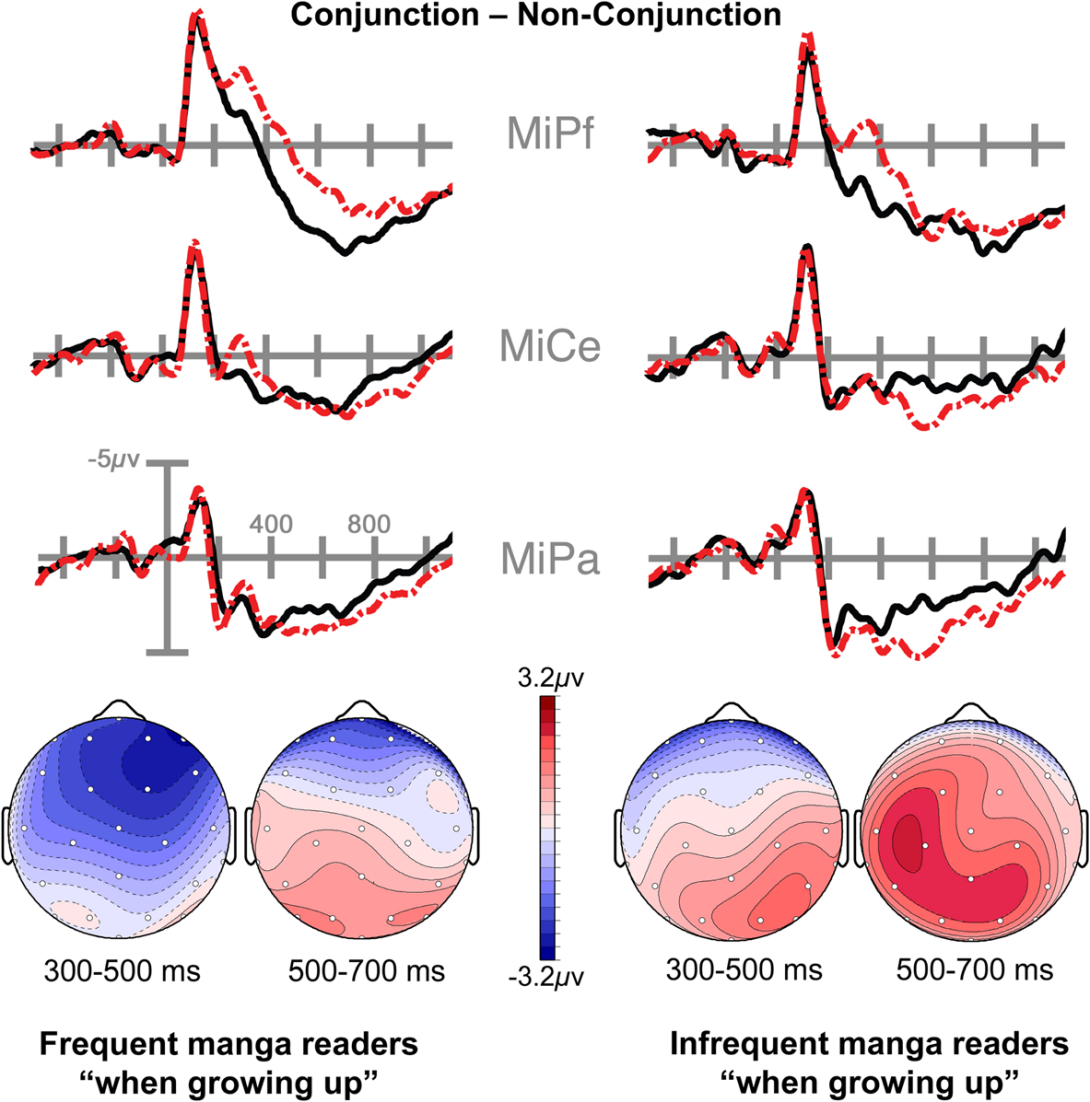
Midline electrode sites and topographic voltage maps representing distribution across the scalp for the difference between Non-Conjunction and Conjunction sequences for frequent and infrequent readers of Japanese manga “while growing up.” *Pa* parietal, *Pf* prefrontal, *Mi* Midline, *Ce* Central

Based on this finding, we again ran our ANOVAs setting Structure (Conjunction vs. Non-Conjunction), Hemisphere, AP Distribution, and Laterality (see Methods) as within-subjects factors, for both the 300–500 ms and 500–700 ms epochs, and adding Fluency as a covariate (i.e., participants’ self-reported measure of manga reading while growing up). In both epochs, omnibus ANOVAs showed a significant four-way interaction between Structure, AP Distribution, Laterality and Fluency (all *F* > 2.6, all *p* < 0.05).Visual inspection showed that the difference between groups manifested in reciprocal modulations of the anterior negativity and posterior positivity. More frequent manga readers showed a more widespread anterior negativity in the 300–500 ms epoch, with a reduced posterior positivity starting in the 300–500 ms epoch and extending into the 500–700 ms epoch. By contrast, less frequent manga readers showed a reduced and focal anterior negativity, with a wider and larger posterior positivity. To illustrate these patterns, we divided participants using a median split into groups of frequent manga readers while growing up (*N* = 14, mean frequency M = 6.57 out of 7) and infrequent manga readers (*N* = 14, M = 3.14) and depict this in Fig. 6.

## Discussion

We assessed two alternative views of visual narrative (sequential image) processing by analyzing ERPs to E-Conjunctions. On both accounts, comprehenders incrementally update an evolving mental model triggered by changes in perceptuo-semantic content from panel to panel. However, the VNG account further posits that visual narrative comprehension involves an additional (grammatical) combinatoric component. Consistent with the VNG framework, but not the canonical semantic-updating account, we also hypothesized that these two visual narrative comprehension components would be modulated by participants’ experience with particular visual narrative constructions (in this case, E-conjunctions), which based on corpus analysis is more prevalent in Japanese manga than American comics (Cohn, 2011, 2013a, in press; Cohn, Taylor-Weiner, et al., 2012b).

### Processing visual narratives

As predicted, at the critical panel, we observed an ERP index of mental model updating in a late (400–900 ms) posterior positivity (Brouwer et al., 2012; Donchin & Coles, 1988). In line with reports of P600s to referential changes in discourse (e.g., Nieuwland & Van Berkum, 2005; Van Berkum et al., 2007), we also found that the P600s were modulated both by conjunctions and referential incongruities. We take the larger positivity to Congruous Conjunctions than Non-Conjunctions as indexing the mental updating process of incorporating each character into a common space, and/or the revision of the narrative structure (Cohn et al., 2014; Cohn & Kutas, 2015), consistent with the reanalyses of syntactic structure in language (Brown & Hagoort, 2000; Brown et al., 2000; Osterhout & Holcomb, 1992) and music (Patel et al., 1998).

The similar P600 amplitudes for Congruous Conjunctions and Incongruous Non-Conjunctions are consistent with equivalent updating processes triggered by an unexpected character change. In the case of Congruous Conjunctions, the panels shift from one character to another while maintaining sequence congruity. In the case of Incongruous Non-Conjunctions, a character, incongruous with the prior expectations established by the sequence, is added to the scene. The even larger P600 to Incongruous Conjunctions indicates that more effortful or substantive updating may be required when character changes co-occur with the need to draw inferences, in this case about a common spatial environment. Such a response may also indicate an interaction between spatial/referential updating and reanalysis for the narrative structure. Overall, the P600 findings accord with ongoing mental updating of referential and spatial information, consistent with both VNG (Cohn, 2014a; Cohn & Kutas, 2015) and models reliant on perceptuo-semantic information (Bateman & Wildfeuer, 2014; Magliano & Zacks, 2011).

As predicted, we also found ERP signs of a process preceding mental updating—namely, an anterior negativity (over prefrontal sites) that was larger for conjunctions than nonconjunctions, regardless of congruity. We take this as an index of combinatorics, which we argue is, like mental updating, part and parcel of visual narrative comprehension. As noted in the introduction, anterior negativities have been seen in response to violations of structural expectations in visual narratives (Cohn et al., 2014; Cohn, Paczynski, et al., 2012a) and syntactic structure in sentences (i.e., the LAN; e.g., Friederici, 2011; Hagoort, 2003; Neville et al., 1991) and music (i.e., the RAN; Koelsch et al., 2005; Patel et al., 1998). Our observed anterior negativity was insensitive to unexpected character changes, consistent with our linking hypothesis that it reflects structural processing (i.e., the conjunction schema) and not updating of semantic information. It seems, then, that panels involved in conjunctions are more structurally costly than those in nonconjunction sequences.

The insensitivity of the anterior negativity to semantics—particularly in contrast to the P600—is important in two respects. First, it shows that, as in sentence processing, anterior negativities are sensitive to aspects of structure, independent of semantics (Münte, Matzke, & Johannes, 1997). A separation between meaning and grammar was also inferred from the insensitivity of the N400 (an index of semantic processing) to narrative structure (Cohn, Paczynski, et al., 2012a). Here we show the reverse: the anterior negativity is insensitive to semantic incongruity.

Second, we take this insensitivity to semantics to mean that this anterior negativity is not an N400 (Sitnikova et al., 2008; West & Holcomb, 2002), with a frontal skew due to overlap with a posterior P600 (Tanner & Van Hell, 2014). If this was the case, we would have expected greater negativity to Incongruous panels than Congruous panels, but this did not occur. Rather, congruity had no influence on the anterior negativity and it was sensitive only to the combinatorial conjunction pattern.

### Cross-cultural variation

Based on corpus analyses which have implied that Japanese manga uses more E-Conjunctions than American comics (Cohn, 2011, 2013a, in press; Cohn, Taylor-Weiner, et al., 2012b), we hypothesized that participants’ differential experience with these comics might modulate visual narrative processes—both mental updating and structural analyses. And, that is what we found. The conjunction effect was modulated by participants’ experience reading Japanese manga while growing up, but not by any other measures of participants’ background comic reading experiences (note also, our *Peanuts* stimuli did not graphically resemble manga). The ERPs of frequent manga readers were characterized by larger anterior negativities, with reduced P600s, while those of infrequent manga readers were characterized by larger P600s with reduced anterior negativities. These findings suggest that comprehenders familiar with E-Conjunction through manga reading are likely to engage in more combinatoric processing, relying on a schematic pattern encoded in memory. Not mutually exclusive to this, the attenuated P600 to experienced readers could suggest an easier time in drawing the spatial inference of the conjunction. In contrast, less frequency of reading manga may invoke more mental updating of semantic information, perhaps compensating for lacking an entrenched combinatoric narrative pattern. Similar tradeoffs between negative responses (N400s) and posterior positivities (P600s) have been observed across individuals in ERP research on sentence processing (Tanner & Van Hell, 2014), albeit not tied to experience with particular constructions.

It is noteworthy that variation along an anterior–posterior axis also appears in neuroimaging research on linguistic experience. Deaf adults who acquired sign language at an early age showed more left anterior neural activation to grammatical judgments than late learners, who showed more posterior activation (Mayberry, Chen, Witcher, & Klein, 2011). Moreover, more posterior activation to signed and verbal languages also characterizes individuals who are younger, have later age of acquisition, and/or are less fluent (Brown et al., 2005; Meyer et al., 2006; Schlaggar et al., 2002). Such findings may suggest that earlier fluency enables more automatic processing in anterior regions (Mayberry et al., 2011). This possibility is consistent with our observation that readers fluent in E-Conjunction use earlier, anterior combinatoric structural processes, with less reliance on posterior mental updating processes. That our participants differ as a function of manga experience specifically “while growing up” may imply “age of acquisition” effects for visual narratives, similar to modulation of sequential image comprehension by both age and exposure to comics (Nakazawa, 2016).

## Conclusions

Altogether, our findings indicate that visual narrative comprehension involves multiple interacting processes: here, updating of a mental model and a combinatorial narrative grammar. Insofar as researchers believe that the same mechanisms operate in the understanding of narratives across domains (Cohn, 2013b; Gernsbacher, 1990; Magliano, Loschky, Clinton, & Larson, 2013), our results raise questions about how specific narrative patterns (like E-Conjunction) align with frequency of those patterns in domains outside of drawn visual narratives, such as discourse and film.

Our results provide further evidence for overlap in neurocognitive processing across domains, such as language, music, and visual narratives (Cohn et al., 2014; Magliano et al., 2015; Patel, 2003). Consistent with the literature, we observed similar electrophysiological markers for the processing of visual narratives and language (Cohn, 2013a; Cohn et al., 2014; Cohn, Paczynski, et al., 2012a). Such parallels are reinforced by our finding that visual narrative comprehension is conditioned by “fluency” in particular visual narrative systems. Because this fluency seems to map onto an anterior–posterior axis it aligns with work on language proficiency, suggesting reliance on domain-general processing that extends beyond the scope of visual narratives and/or language. Thus, studying visual narratives, and experience with them, can potentially inform our broader understanding of cognitive processes which may otherwise be viewed as domain specific.

Finally, such findings question the belief that sequential images are uniformly processed across individuals. Given that even basic sequential image processing requires exposure to visual narratives (e.g., Byram & Garforth, 1980; Fussell & Haaland, 1978), these results suggest that such fluency follows acquisition of culturally diverse structures, which in turn modulate understanding. Such variability raises questions about the validity of the assumption that sequential images make universally accessible stimuli in experimental tasks and education materials, and indicate the need for further research on aspects of fluency in and across these visual languages.

## Additional file

Additional file 1: Available data. (CSV 53 kb)

## Abbreviations

CC: Congruous Conjunction
CNC: Congruous Non-Conjunction
IC: Incongruous Conjunction
INC: Incongruous Non-Conjunction
